# Caregiver recruitment and engagement: identifying best practices and strategies for including family caregivers of veterans in health services research

**DOI:** 10.1186/s12913-025-13368-3

**Published:** 2025-10-06

**Authors:** Victoria Ngo, Luci K. Leykum, Andrea F. Kalvesmaki, Stuti Dang, Polly H. Noël, Mary Jo Pugh, Roxana E. Delgado, Erin D. Bouldin, Julie Parish, Ranak B. Trivedi, Elizabeth Dole Center of Excellence for Veteran and Caregiver Research Team

**Affiliations:** 1 Department of Veterans Affairs, Elizabeth Dole Center of Excellence for Veteran and Caregiver Research, Washington, DC USA; 2 Center for Innovation to Implementation, Veterans Affairs Palo Alto Health Care System, 795 Willow Road (152-MPD), Menlo Park, CA 94025 USA; 3 Stanford Health Policy, Stanford School of Medicine and Freeman Spogli Institute for International Studies, Stanford University, Encina Hall, 616 Jane Stanford Way, Stanford, CA 94305 USA; 4 New England Geriatric Research, Education, and Clinical Center (GRECC), Department of Veterans Affairs Bedford Healthcare System, 200 Springs Road, 18-182B, Bedford, MA 01730 USA; 5 The South Texas Veterans Health Care System, 7400 Merton Minter, San Antonio, TX 78229 USA; 6 Department of Internal Medicine, Dell Medical School, The University of Texas, 1601 Trinity Street, Bldg. B, Stop Z0900, Austin, TX 78712 USA; 7 Informatics, Decision-Enhancement and Analytic Sciences Center (IDEAS) Center, Veterans Affairs Salt Lake City Health Care System, 500 Foothill Drive Bldg. 182, Salt Lake City, UT 84148 USA; 8 Department of Internal Medicine, Division of Epidemiology, University of Utah School of Medicine, 295 Chipeta Way, Salt Lake City, UT 84108 USA; 9 Miami Veterans Affairs Healthcare System, Geriatric Research, Education and Clinical Center (GRECC), 1201 N.W. 16Th Street, W. GRECC (11GRC), Miami, FL 33125 USA; 10 Division of Geriatrics and Palliative Medicine, Department of Medicine, University of Miami Miller School of Medicine, 1600 NW 10Th Avenue, Suite 1149, Miami, FL 33136 USA; 11 Department of Family and Community Medicine, University of Texas Health Science Center, 7703 Floyd Curl Drive, MC 7843, San Antonio, TX 78229 USA; 12 School of Nursing, The University of Texas Health at San Antonio, 7703 Floyd Curl Drive, San Antonio, TX 78229 USA; 13 Harte Research Institute, Texas OneGulf Center of Excellence, 6300 Ocean Drive, Unit 5869, TX 78412 Corpus Christi, USA; 14 Division of Public Mental Health and Population Sciences, Department of Psychiatry and Behavioral Sciences, Stanford University, 401 Quarry Road, Stanford, CA 94305 USA

**Keywords:** Recruitment strategies, Study participants, Caregiving research, Participant engagement, Target population

## Abstract

**Background:**

Family caregivers are integral to the care of patients and possess important perspectives regarding improving healthcare delivery. Yet, caregivers are seldom engaged in research in part because of challenges recruiting and retaining them. We sought to identify best practices for recruiting and retaining caregivers in health services research, drawing from researcher experiences in the Department of Veterans Affairs (VA).

**Methods:**

We identified VA Health Systems Research (HSR) funded studies that focused on caregivers of Veterans by reviewing ongoing and completed projects from 2017 through 2024. “Caregiver” was defined as family members and/or friends who helped Veterans manage their health issues. We interviewed principal investigators and study team members to characterize their recruitment and retention practices. We further examined their studies’ published methods and results to summarize the breadth of strategies used, and to identify those that were most effective based on informants’ impressions of what worked.

**Results:**

Seventeen research team members from 11 studies at six sites participated. Studies were observational (8 studies, *n* = 4183), experimental (2 studies, *n* = 237), and implementation-focused (1 study, *n* = 435), and included 4855 caregivers. All studies utilized multiple recruitment approaches, most commonly directly approaching potential participants, utilizing administrative data, and advertising. The most successful practices were: (1) directly approaching Veterans and caregivers; (2) recruiting through clinics; (3) reviewing registries of prior study participants; and (4) using administrative data to identify potential candidates. Flexible, virtual scheduling and individualized participant activities utilizing multiple participation modalities were helpful for caregiver retention.

**Conclusions:**

Successfully recruiting and retaining caregivers requires multiple strategies, and researcher flexibility. Health services researchers should prioritize direct outreach, outreach through trusted clinicians, and establishing registries to optimize efforts. Researchers should also use methods that allow for flexible, individualized participation to maximize retention. Enhancing caregiver-focused research through optimizing caregiver engagement will ultimately improve the evidence base necessary to inform care delivery and policy.

**Supplementary Information:**

The online version contains supplementary material available at 10.1186/s12913-025-13368-3.

## Introduction

Approximately 53 million American, unpaid, family caregivers care for adults living with chronic conditions [1]. Caregivers assist with activities of daily living (ADLs; e.g., bathing, dressing), instrumental ADLs (IADLs; e.g., meal planning, paying bills), care coordination, socialization, and other needs [2]. Persons who have caregiver support are less likely to be hospitalized or require institutional care than persons who do not, underscoring the caregivers’ role and substantial impact on care outcomes [3, 4].

Improved care recipient outcomes may occur at the expense of caregivers’ well-being. Caregivers often experience worse physical and mental health outcomes, and are more likely than non-caregivers to perceive their own health as poor [5–7]. The tension between the increased need for caregiving and worsened caregiver health outcomes is a public health concern [8]. Recent legislation, such as the Recognize, Assist, Include, Support, and Engage Family Caregivers (RAISE) Act (Pub L No. 115–119) [9] and the Biden Harris Executive Order on Increasing Access to High-Quality Care and Supporting Caregivers [10], focuses on developing an evidence-based, national family caregiving strategy. Effective approaches for identifying and engaging caregivers are vital to these efforts.

The Department of Veterans Affairs (VA) is a leader in integrating family caregivers into Veteran care, and in funding and conducting caregiver-related research. To support these efforts, VA Health Systems Research (HSR; previously Health Services Research and Development, HSR&D) requested the co-authors identify best practices for recruiting caregivers. We sought to: (1) describe recruitment and engagement strategies used in health services research studies funded by VA; and (2) identify best practices and lessons learned in recruiting and retaining family caregivers.

## Methods

We identified studies funded by the VA HSR (formerly HSR&D) that included caregivers as research participants, by reviewing ongoing and completed HSR-funded studies from 2017 through 2024. “Caregiver” was defined as family members and/or friends who helped Veterans manage their health issues or chronic conditions.

To catalog and assess the recruitment and retention strategies utilized, we contacted the principal investigators of each study, and asked them to identify research personnel best able to report on recruitment and retention experiences. To minimize potential respondent bias, we standardized our interviews, utilizing a consistent set of questions asked by two team members. We also triangulated responses with reported recruitment outcomes. In addition to interviews, we also reviewed published manuscripts to assess recruitment approaches and numbers of recruited caregivers. Two research team members conducted interviews and collected data. All study team members had expertise in family caregiving and experience conducting key informant interviews and coding qualitative data.

We developed questions specific to this assessment (see Supplementary File 1). We asked research personnel participants: (1) What recruitment strategies did you use?; (2) What strategies worked best in terms of recruiting the greatest number of caregivers?; (3) What challenges did you experience in ongoing caregiver engagement in research activities?; (4) What strategies did you use to keep caregivers engaged in the research over time? Interviews were conducted via phone with field notes taken.

This work was part of an ongoing program evaluation undertaken by the Elizabeth Dole Center of Excellence for Veteran and Caregiver Research. This work was reviewed by the Institutional Review Board of the University of Texas Health Science Center at San Antonio and determined to not be regulated research. It was also reviewed as non-regulated research by the Research & Development Committee of the South Texas Veterans Health Care System. Because this work was not considered regulated research, participants’ written informed consent was not required. However, participants provided verbal consent at the start of each interview.

### Data analysis

All members of the research team had experience conducting qualitative research. Three members analyzed responses and publications, independently identifying common themes in information provided regarding recruitment and ongoing engagement/retention. These team members then met to discuss the themes, agreeing on categorization and description of recruitment strategies and approaches to maximize caregiver participation and their relative effectiveness. Our assessment of effectiveness was driven by informants’ perceptions of what worked.

## Results

We identified eleven studies that were funded by VA HSR and interviewed representatives of all eleven. Eleven investigators, two project managers, one study coordinator, one research assistant, and two research fellows provided information. From the 17 respondents who were interviewed, 1–2 respondents shared their experience for each study. A total of 4,855 caregivers were recruited across 11 studies conducted at six VA sites: San Antonio, TX; Palo Alto, CA; Salt Lake City, UT; Miami, FL; Durham, NC; Cleveland, OH. Study designs included qualitative studies utilizing interviews and focus groups (5 studies, *n* = 92); mixed methods studies utilizing web-, telephone-, and paper-based surveys (4 studies, *n* = 4526); interventions (2 studies, *n* = 237); and a national implementation study (1 study, *n* = 435). Studies recruited caregivers alone (8 studies, *n* = 1269) or Veteran-caregiver dyads (3 studies, *n* = 3586) (see Supplementary File 2). Recruitment for all studies at least partially overlapped with the COVID-19 pandemic.

### Recruitment strategies

The most common recruitment strategies were: directly approaching Veterans and caregivers through clinics and front-line clinicians (6/11); utilizing administrative data for targeted outreach (4/11); advertising (4/11); directly asking Veterans about their caregivers (3/11); partnering with national and local community-based organizations (3/11); recruiting from registries of previous study participants (2/11); and recruiting through local VA program offices (2/11) (Table 1). Studies typically used more than one strategy in complementary ways. For example, administrative data were used to identify Veterans based on their health conditions, who were then contacted regarding the presence of a caregiver. We described these strategies, their overlap, and their perceived effectiveness.

**Table 1:** Caregiver recruitment approaches utilized by research teams at the VA

Recruitment strategy	(N studies), % of studies	Specific approaches
Recruiting through clinics and front-line clinicians	(6/11), 55%	• Flyers in clinical locations • Provided information to clinicians to share with caregivers • Clinician referral • Invitation letter to potential caregiver participants • Provided VA clinicians with a study overview and referral instructions • Notification to clinical services at VA • Sent letters to patients
Identifying potential participants using administrative data and subsequent outreach	(4/11), 36%	• Veterans were identified using the VA high need, high risk list of those at risk for institutional care • EHR-driven recruitment approach: ◦ Selected Veterans with EHR referrals to five home- and community-based services ◦ Identified potential live-in caregivers by looking for a next-of-kin with the same address as the Veteran • Identified veterans with a diagnosis of dementia or memory loss or who were prescribed anti-dementia 5 medications, followed by chart review
Advertising	(4/11), 36%	• Flyers and newsletters • Social media and websites
Directly asking Veterans and their care recipients	(3/11), 27%	• Veterans were mailed information and received follow-up phone calls to invite them and their caregivers to participate • Called to confirm the presence of caregiver • Confirmed caregiver eligibility via telephone screening
Partnering with national and local community-based organizations	(3/11), 27%	• Community-based partner recruitment approach • Blogs and Podcasts • Caregiver community advisory group • Emails from organizations serving caregivers of Veterans (e.g., Elizabeth Dole Foundation, Military and Veteran Caregiver Network, Hearts of Valor)
Recruiting from registries of previous study participants	(2/11), 18%	• Mailed outreach to Veterans and phone contact with caregivers • Veteran survey also had a question asking them to identify up to 3 additional caregivers and provide their addresses so Caregiver surveys could be mailed to them directly. Identified caregivers were sent a survey by mail
Recruiting through local program offices	(2/11), 18%	• The Caregiver Support Program at some facilities provided information to caregivers of Veterans who were enrolled at their facility • Connected with Veteran Service Organizations and presented to their teams

#### Strategy 1: Recruiting through clinics and front-line clinicians

Most studies (55%) recruited family caregivers through clinics and front-line clinicians [11–15]. Front-line clinicians served as trusted points of contact. The following approaches were used: (1) sending informational materials to clinicians to disseminate; (2) placing informational materials in waiting rooms or check-in desks; (3) having a study team member approach Veterans and/or caregivers in waiting areas. One researcher noted targeting clinicians who cared for Veterans at high-risk of needing long-term services. Approaching clinical leaders and supervisors prior to recruitment was recommended.

#### Strategy 2: Identifying potential participants using electronic health record (EHR) or administrative data and subsequent outreach

Thirty-six percent (36%) of studies identified potential participants using EHR or administrative data [16–19]. Because family caregiver contact information was not available in a standardized location, researchers described challenges in identifying caregiver information within the EHR. Common places included: (1) next of kin, (2) emergency contact, or (3) social worker notes. In one study, investigators looked for next of kin with the same address as the Veteran to identify potentially co-residing caregivers. In another, the team sent letters to next of kin of Veterans seen in Women’s Health Clinics. Letters were signed by the PI and clinicians, and follow-up telephone calls were made to determine interest and eligibility. One site identified Veterans based on referrals to specific home- and community-based services and called to determine the presence and interest of potential caregivers.

#### Strategy 3: Advertising to caregivers

Advertising information was sent to potential participants through mail or social media and websites (36%) [12–14, 19, 20]. Mail invites required printing and mailing costs, as well as having contact information for specific individuals or patient populations. Social media and website advertising did not identify potential participants through general profile descriptions. Hence, digital displays or web-based notifications at VAMCs, social media posts (e.g., Facebook, Twitter), advertising via partner organization webpages or through groups dedicated to military caregiving, were low-cost alternatives. Several investigators used VA caregiver-specific websites, such as the VA National Caregiver Research Interest Group.

#### Strategy 4: Directly asking Veterans for caregiver information

Direct contact with Veterans was the most effective way to identify caregivers; 27% of studies used this strategy [12, 14, 19]. Researchers successfully approached Veterans and their family caregivers at clinic appointments (see Strategy 1) or community events. For a large, multisite survey, caregiver surveys were mailed to Veterans, who were asked to give the survey to the person most involved in their care [19]. These letters included QR codes to facilitate web-based participation.

#### Strategy 5: Partnering with national and local community-based organizations

Collaborating with community-based organizations provided another avenue to recruit caregivers (27%) [12–14, 20]. To recruit youth in caregiving households, researchers partnered with a range of local Veteran and Military family-serving organizations who passed study materials on to their constituents. This partnership strategy had mixed success, as organizations had varying degrees of ongoing contact with caregivers. One study utilizing this strategy was not successful.

Partnerships were contingent on building and maintaining trusting relationships with community partners. Since relationships took time to develop, respondents recommended developing and maintaining relations before and outside of individual research projects. Partnering with the Veteran Engagement Workgroup or local Veteran Advisory Boards was a suggested first step. The VA Veteran Engagement Workgroup is a collaborative that actively involves Veterans in the research process by soliciting their input to improving the design, execution, and dissemination of Veteran-relevant research [21]. Veteran Advisory Boards are local groups composed of Veterans, family members, and community representatives who work with VA leadership to provide insight, feedback, and advice on policies relevant to veteran healthcare delivery [22].

#### Strategy 6: Recruiting from registries of previous study participants

Contacting caregivers who had previously participated in VA research studies was effective. Two studies (18%) recruited from registries of former participants willing to be contacted. These registries were possible because investigators previously asked in former studies whether study participants agreed to be contacted for future research studies [14]. Investigators suggested proactively asking participants about willingness to be contacted for future studies as groundwork for future research.

#### Strategy 7: Recruiting through local program offices

Recruitment also occurred through local VA program offices (18%) [13, 14]. Because these offices often had direct working relationships with caregivers, they were an effective outreach resource. Several researchers worked with their local Caregiver Support and Geriatrics and Extended Care (GEC) Programs to share study information. Program offices offer a range of services for different groups of Veterans, so reaching different types of caregivers was possible.

### Combinations of recruitment approaches

All studies used combinations of recruitment approaches. The most effective combinations included using administrative EHR data to identify potential participants and approaching caregivers directly through clinics and front-line clinicians.

### Approaches for sustaining engagement and retention of caregivers

Successfully retaining caregivers in research and interventions required proactively identifying and addressing barriers to participation. Programs with fixed participation times made participation difficult because of caregivers’ multiple responsibilities. This required flexibility in conducting study activities. Examples of adaptations included: (1) Contacting outside of regular working hours; (2) Splitting research activities into shorter periods; (3) Rescheduling planned activities based on a caregiver’s change in availability. Accommodating caregiver schedules maximizes the likelihood of successful retention.

Travel and transportation were often cited as barriers, sometimes making virtual participation more feasible. Conversely, some groups of caregivers had limited access to technology or Internet, or limited comfort with virtual modalities. Researchers noted that the following strategies may be helpful: (1) Offering both in-person and virtual participation options; (2) Eliciting caregivers’ preferences regarding participation modality; and (3) Changing participation method as needed.

Because of caregivers’ scheduling challenges, researchers shared that individual research activities may be more feasible than group activities. Some researchers expanded caregiver data collection strategies from only focus groups to include individual interviews and small group interviews. This enabled accommodating individual preferences for participation and minimized the impact of last-minute scheduling changes.

## Discussion

VA researchers used multiple strategies for recruitment and retention of family caregivers of Veterans in health services research, including outreach through clinics, program offices, advertising, and use of administrative data, typically in combination. The most effective approaches involved direct contact with the Veteran and caregiver. Because consistent documentation of caregivers in the electronic health record would facilitate this direct contact, we recommend standardizing the documentation of caregiver information the electronic health record. Creating registries of caregivers interested in research participation is also effective but requires planning. One way to do this is to ask participants if they can be contacted for future research and document consent. Early career researchers could rely on existing registries as they build their own. Finally, developing relationships with front-line clinicians, program offices, and other recruitment partners also takes time and requires trust [23, 24].

Similar to prior studies [25, 26], we found that family caregivers experience barriers to longitudinal research participation. Finding time to participate on top of caregiving responsibilities is a key obstacle. This was best addressed by using multiple strategies, including flexibility in the timing of participation in research activities and participation modality.

We assessed experiences of a national group of researchers conducting different types of research across diverse populations of caregivers. While our scope was limited to VA health services research, our insights are likely generally relevant. Because the VA has established a number of services and supports for caregivers, its infrastructure for identifying and recruiting caregivers may be more robust than in non-VA settings. Assessing strategies for recruitment and retention of caregivers in research studies in non-VA settings is important, particularly as health systems and community organizations increase their engagement with family caregivers.

### Limitations

We relied on the recollections of study personnel regarding relative effectiveness of different approaches. While we took steps to mitigate the bias as noted in Methods, recollections and study personnel’s individual perceptions may or may not accurately reflect the thoughts, feelings, and experiences of the caregivers in their studies. Many studies did not document the numbers of caregivers recruited through each strategy, so we were unable to quantitatively compare the effectiveness of each strategy. Systematically assessing the relative effectiveness of different strategies for different populations would also provide valuable information. Finally, we did not assess the cost of the various recruitment strategies. Differences in time or personnel required for different strategies could influence the feasibility of their use. While we do not have the data to analyze this across the multiple studies, as an example, for the Trivedi et al. [14] study, 2.5 Full-time Equivalents (FTEs) recruited 23 caregivers over a duration of 7 months. FTE is a human resource unit of measurement used to express the workload of an employee in terms of a full-time position. For example, an FTE of 1.0 means a full-time employee.

## Conclusions

Including caregivers in research is vital for effectively supporting this hidden workforce. Our findings can guide recruitment practices for future research initiatives and provide specific implementation strategies for successful retention. Trust is critical for successful engagement but is a long-term commitment that goes beyond individual research studies. Health systems should invest in these connections to support research.

## Supplementary Information

Supplementary Material 1.

Supplementary Material 2.

## Data Availability

No datasets were generated or analysed during the current study.

## Abbreviations

VA: Department of Veterans Affairs
HSR: Health Systems Research
ADL: Activities of Daily Living
IADL: Instrumental Activities of Daily Living
HER: Electronic Health Record
GEC: Geriatrics and Extended Care
FTE: Full-time Equivalent
