# Supplementary material for: Caregiver recruitment and engagement: identifying best practices and strategies for including family caregivers of veterans in health services research
Source: BMC Health Serv Res. 2025 Oct 6;25:1316. doi: 10.1186/s12913-025-13368-3 (PMC12502154; doi:10.1186/s12913-025-13368-3)
Supplement: Supplementary file 1 — Supplementary Material 1. [file 12913_2025_13368_MOESM1_ESM.pdf]

Supplementary File 1. Interview Guide for Personnel Engaging in Caregiving Research

---

Department of Veterans Affairs Researchers,

As part of our ongoing program evaluation at the Elizabeth Dole Center of Excellence for Veteran and Caregiver Research, we would like to learn more about some of the recruitment strategies you have used to identify and include caregivers in research at the Department of Veterans Affairs.

Please help answer the following questions to the best of your ability:

1. What recruitment strategies did you use?

2. What strategies worked best in terms of recruiting the greatest number of caregivers?

3. What challenges did you experience in ongoing caregiver engagement in research activities?

4. What strategies did you use to keep caregivers engaged in the research over time?

[Thank you for taking your time to answer these questions]
