# Supplementary material for: Caregiver recruitment and engagement: identifying best practices and strategies for including family caregivers of veterans in health services research
Source: BMC Health Serv Res. 2025 Oct 6;25:1316. doi: 10.1186/s12913-025-13368-3 (PMC12502154; doi:10.1186/s12913-025-13368-3)
Supplement: Supplementary file 2 — Supplementary Material 2. [file 12913_2025_13368_MOESM2_ESM.pdf]

Supplementary File 2. Caregiver Studies at the VA that informed our experiences

Name	Methods	Strategy number	Recruitment Strategies	Recruitment Goal	Participants (N)	Recruitment Outcome (%)
Caregiver perceptions of home and community-based services[16]	Telephone interviews and both in-person as well as virtual focus groups	2	• Identified potential participants using administrative data and subsequent outreach<br>○ Veterans were identified using the VA high need, high risk list of those at risk for institutional care<br>○ Veterans were mailed information and received follow-up phone calls to invite them and their caregivers to participate.	8-10 per group	24 caregivers, 34 Veterans, 39 leaders	240%
Caregiver experience with Tele-dementia[11]	Qualitative grounded theory using Semi-structured interviews	1	• Recruited through clinics and front-line clinicians<br>○ Clinician referral	30	30	100%
Implementation of helping invested family members improve veteran experiences (iHI-FIVES)[17]	Telephone survey	2	Identified potential participants using administrative data and subsequent outreach<br>• EHR-driven recruitment approach:<br>○ Selected Veterans with EHR referrals to five home- and community-based services<br>○ Called to confirm the presence of caregiver	450	813 Veterans screened<br>435 caregivers enrolled	97%
The impact of the COVID-19 pandemic on Veteran caregivers (VetCORE)[12]	Mixed methods observational	1, 3, 4, 5	• Partnered with national and local community-based organizations<br>○ The Caregiver Support Program at some facilities provided information to caregivers of Veterans who were enrolled at their facility.<br>• Recruited through clinics and front-line clinicians<br>• Directly asked Veterans and their care recipients<br>○ Asked Veterans and caregivers in the geriatrics clinic and home-based primary care to participate<br>• Advertised<br>○ Provided flyers with the QR code to respond to the survey	50 Survey, 30 interview	54 Survey, 28 interview	93%
Supporting Caregivers of Rural Veterans Electronically (SCORE)[21]	Multi-site RCT: intervention of technology or telephone-delivered support group	2	• Identified potential participants using administrative data and subsequent outreach<br>○ Identified veterans with a diagnosis of dementia or memory loss or who were prescribed antidementia medications, followed by chart review<br>○ Identified potential live-in caregivers by looking for a next-of-kin with the same address as the Veteran<br>○ Provided VA clinicians with a study overview and referral instructions<br>○ Invitation letter sent to potential caregiver participants.	200 Salt Lake City; 150 Other locations	186 Salt Lake City; 45 Other locations	93%
Caregiver distress phenotyping and suicide ideation[13]	Web-based survey via an electronic link	1, 3, 5, 7	• Partnered with national and local community-based organizations<br>• Recruited through local program offices<br>• Recruited through clinics and front-line clinicians<br>• Advertised<br>○ Electronic link disseminated through:<br>▪ Social media<br>▪ Newsletters<br>▪ Flyers<br>▪ Emails from organizations serving caregivers of Veterans (e.g., Elizabeth Dole Foundation, Military and Veteran Caregiver Network, Hearts of Valor)<br>▪ Blogs and Podcasts	500	458	92%

			▪ Caregiver community advisory group			
Identifying caregivers' unmet needs for home and community-based services[14]	Qualitative grounded theory using Semi-structured interviews	1, 3, 4, 5, 6, 7	• Recruited through clinics and front-line clinicians<br>o Flyers in clinical locations<br>o Notified clinical services at VA<br>• Advertised<br>o Social media and website<br>• Directly asked Veterans and their care recipients<br>• Recruited through registries of previous study participants<br>o Mailed outreach to Veterans and phone contact with caregivers<br>• Recruited through local program offices<br>o Connected with VSO and present to their teams<br>• Partnered with national and local community-based organizations<br>o Partnered with Caregiver Support Program (CSP)	30	23	77%
Advancing Science of Population-based measures of Independence to help Veterans REmain at Home (ASPIRE)[15]	Qualitative in-person focus groups	1	• Recruited through clinics and front-line clinicians<br>o Clinician referral from patients receiving services from geriatrics primary care, home-based primary care, inpatient social work, homemaker and home health aide care, or Caregiver Support Program<br>o Sent letters to patients<br>o Confirmed caregiver eligibility via telephone screening	16-20	14	70%
HERO CARE Survey of unmet Veteran and caregiver needs[19]	Paper and web-based survey	2, 3, 4	• Identified potential participants using administrative data and subsequent outreach<br>o Veterans identified through VA high need, high risk list of those at risk for institutional care<br>• Advertised<br>• Directly asked Veterans and their care recipients<br>o Veterans mailed survey packets, which included a Veteran and a caregiver survey for Veterans to give to the person most involved in their care. All surveys had a bitly code for respondents to respond online.<br>o Veteran survey also had a question asking them to identify up to 3 additional caregivers and provide their addresses so Caregiver surveys could be mailed to them directly. Identified caregivers were also sent a survey by mail.	20,000 Veterans were mailed a Veteran and a caregiver survey	3,579 caregivers, 8056 Veterans	18%
Caregiver skills training	3- hour training and post-training standardized assessment	1, 6	• Recruited through clinics and front-line clinicians<br>o Flyers in clinical locations<br>o Provided information to clinicians to share with caregivers<br>• Recruited through registries of previous study participants	100	6	6%
Living in a Veteran HomE (LIVE)[20]	Mixed-method observational	3, 5	• Partnered with national and local community-based organizations<br>o Community based partner recruitment approach: partners provided flyers and information to their members via physical postings, social media, and websites.<br>• Advertised<br>o Website with study information that partners could disseminate to interested families.	100 youth caregivers, 100 parents/ guardians	1 youth caregiver and 1 parent	1%

Strategy 1: Recruiting through clinics and front-line clinicians
Strategy 2: Identifying potential participants using electronic health record (EHR) or administrative data and subsequent outreach

Strategy 3: Advertising to caregivers
Strategy 4: Directly asking Veterans for caregiver information
Strategy 5: Partnering with national and local community-based organizations
Strategy 6: Recruiting from registries of previous study participants
Strategy 7: Recruiting through local program offices
